# The hyperimmunoglobulin E syndrome - clinical manifestation diversity in primary immune deficiency

**DOI:** 10.1186/1750-1172-6-76

**Published:** 2011-11-15

**Authors:** Aleksandra Szczawinska-Poplonyk, Zdzislawa Kycler, Barbara Pietrucha, Edyta Heropolitanska-Pliszka, Anna Breborowicz, Karolina Gerreth

**Affiliations:** 1 Department of Pediatric Pneumonology, Allergology and Clinical Immunology, Poznan University of Medical Sciences, 27/33 Szpitalna Street, 60-572 Poznan, Poland; 2 Department of Immunology, The Children's Memorial Health Institute, 20 Dzieci Polskich Street, 04-736 Warsaw, Poland; 3 Department of Pediatric Dentistry, Poznan University of Medical Sciences, 70 Bukowska Street, 60-812 Poznan, Poland

## Abstract

The hyper-IgE syndromes are rare, complex primary immunodeficiencies characterized by clinical manifestation diversity, by particular susceptibility to staphylococcal and mycotic infections as well as by a heterogeneous genetic origin. Two distinct entities - the classical hyper-IgE syndrome which is inherited in an autosomal dominant pattern and the autosomal recessive hyper-IgE syndrome have been recognized. The autosomal dominant hyper-IgE syndrome is associated with a cluster of facial, dental, skeletal, and connective tissue abnormalities which are not observable in the recessive type. In the majority of affected patients with autosomal dominant hyper-IgE syndrome a mutation in the signal transducer and the activator of the transcription 3 gene has been identified, leading to an impaired Th17 cells differentiation and to a downregulation of an antimicrobial response. A mutation in the dedicator of the cytokinesis 8 gene has been identified as the cause of many cases with autosomal recessive hyper-IgE syndrome and, in one patient, a mutation in tyrosine kinase 2 gene has been demonstrated. In this paper, the authors provide a review of the clinical manifestations in the hyper-IgE syndromes with particular emphasis on the diversity of their phenotypic expression and present current diagnostic guidelines for these diseases.

## Introduction

The hyper-IgE syndrome (HIES) was first described in 1966 by Davis, Wedgwood and Schaller [1]; the authors perceived the similarity of severe dermatitis associated with "cold" abscesses with the disease attributed to the prophet Job and hence designated it "Job's Syndrome". In 1972, Buckley and colleagues reported infectious complications in two children who presented with severe chronic dermatitis, coarse faces, and an increased concentration of serum immunoglobulin E; hence these manifestations were termed "Buckley's Syndrome" [2]. Further investigations revealed that increased IgE concentrations and defective neutrophil chemotaxis [3] are recognized in Job's syndrome as well as in Buckley's syndrome, being the same disease entity. In the 1970s, a manifestation of the immune defect in HIES resulted in its inclusion in the group of primary immunodeficiency diseases by Hill et al [4], and the term "Hyper-IgE Recurrent Infection Syndrome" (HIERIS) as proposed by Buckley was also accepted [5]. Extensive reviews of the syndrome were presented in 2000 by Erlewyne-Lajeunesse [6] and in 2005 by Grimbacher and colleagues [7]; furthermore, discussions on the disease chaired by Freeman and Holland have been recently published [8,9].

Although the first data concerning the prevalence of hyper-IgE syndrome referred only to the Caucasian race, further reports indicate its occurence among the Asian and African populations [10,11]; the syndrome occurs in equal frequency among males and females.

Several manifestations of the hyper-IgE syndrome consist of a clinical symptomatology of related diseases, leading to diagnostic difficulties, particularly in young patients and in atypical less severe cases [12] and the diagnosis of pediatric hyper-IgE syndrome is a compilation of symptoms expressed in the later years of patient's life [7].

The hyper-IgE syndrome is a complex immune deficiency with diverse clinical manifestations and heterogeneous genetic origins [13]. Recent studies have demonstrated that hypomorphic mutations in the signal transducer and the activator of transcription 3 (*STAT3*) gene result in the classical multisystemic, autosomal dominant form of HIES, associated with facial, dental, skeletal, and connective tissue abnormalities [14-16]. A *STAT3 *mutation results in a defective multiple cytokine signal transduction, including interleukin (IL)-6 and IL-22, leading to impaired Th17 function and thus explaining the susceptibility to infections in HIES.

In 2004 Renner et al [17] reported an autosomal recessive form of the hyper-IgE syndrome, sharing common features with autosomal dominant HIES, such as hyperimmunoglobulinemia E, susceptibility to staphylococcal infections and cutaneous lesions. However, a different infection profile, a high rate of neurological complications, as well as frequently reported autoimmunity and malignancy, suggest a distinct disease entity. Initially, in a single patient with autosomal recessive HIES, a null mutation in the tyrosine kinase 2 (*TYK2*) gene was identified. The Tyk2 deficiency is responsible for both innate and adaptive impaired immune responses due to defective cytokine signal transduction pathways which depend on interferon (IFN)-α, IL-6, IL-10, IL-12, and IL-23 [18]. In many, although not all cases of autosomal recessive HIES, homozygous mutations of dedicator of cytokinesis gene (*DOCK8*) has been demonstrated, leading to the disruptive production of a protein involved in the regulation of the actin skeleton [19].

## Clinical presentation

### Autosomal dominant HIES

**Figure 1:**
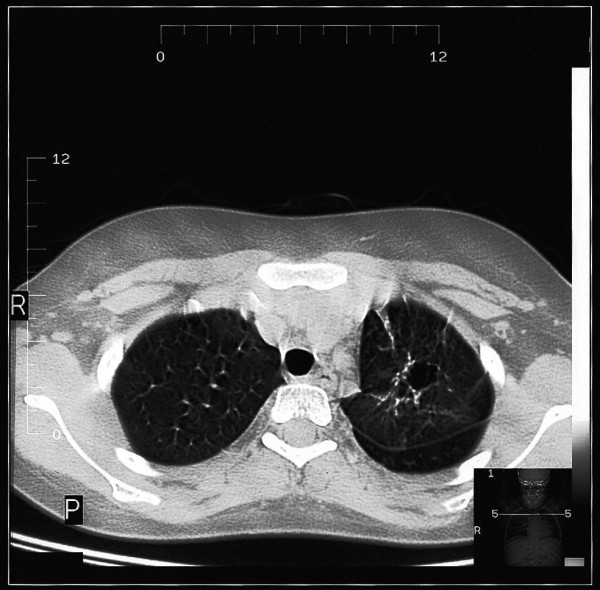
**Fine nodules, interstitial infiltrations and a cystic lesion on chest high-resolution computed tomography (HRCT) in a child with HIES**.

**Figure 2:**
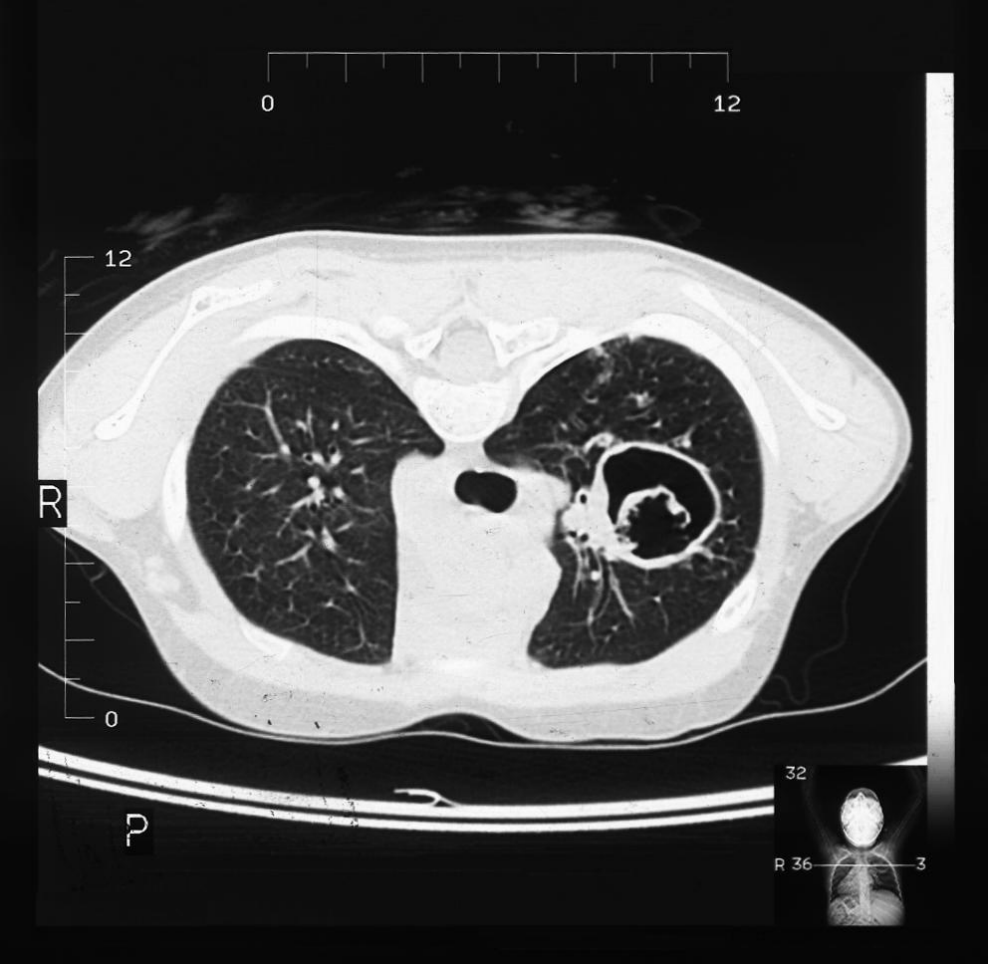
**High-resolution computed tomography of the chest in a child with HIES, showing aspergilloma in a postinflammatory cavity**.

The clinical triad of symptoms found generally in 75% of all cases of AD-HIES and in 85% of patients over 8 years old includes: 1) recurrent staphylococcal abscesses, 2) recurrent airway infections, 3) increased concentration of immunoglobulin E in serum [7]. It has been stressed in the literature that neonatal rash is typically the first clinical manifestation of the hyper-IgE syndrome [20,21]. Interestingly, although chronic dermatitis in the hyper-IgE syndrome is traditionally described as eczema, it is doubtful if this rash really presents as atopic dermatitis [22-24], particularly given that skin biopsies reveal eosinophilic infiltration related to that observed in eosinophilic folliculitis [25]. Skin infections also occur frequently - furunculosis and cellulitis may be observed early on in infancy. "Cold" abscesses, typically observed in patients who are not on antibiotic prophylaxis, are pathognomonic for the hyper-IgE syndrome, but are not necessary for a definitive diagnosis [26,27]. Severe recurrent respiratory infections are usually caused by *Staphylococcus aureus*, including *MRSA *[28] and, less frequently, by *Haemophilus influenzae *and *Streptococcus pneumoniae*. Pneumonias are typically complicated by lung abscesses [29], bronchiectases, bronchopleural fistulas and the formation of pneumatocele [6,30] (Figure 1). These bronchopulmonary lesions are predisposing factors for colonization by opportunistic microorganisms such as *Pseudomonas aeruginosa *and *Aspergillus fumigatus*. The latter can lead not only to invasive aspergillosis requiring intensive therapy, but also to the formation of aspergilloma (Figure 2). Pulmonary sequelae lead invariably to the development of chronic respiratory insufficiency and are the main cause of mortality in HIES. Hemoptysis complicating lung abscess and cystic lung disease are the next common cause of death in HIES reported by Freman et al [31]. Upper airway infections manifest as paranasal sinusitis, exsudative otitis media [32], otitis externa and mastoiditis. In approximately 80% of all cases, mycotic infections of the skin and mucous membranes with *Candida albicans *and other fungal strains may coexist [7]. There are also reports concerning *Pneumocystis jiroveci *infection [33,34], cryptococcosis [35,36], histoplasmosis [37-39], disseminated pulmonary candidiasis [40], *Mycobacterium intracellulare *and *Nocardia infection *[12], as well as post-BCG vaccination complications [12,41,42]. In the autosomal dominant form of hyper-IgE syndrome, infections with herpes simplex virus are relatively infrequent [43].

**Figure 3:**
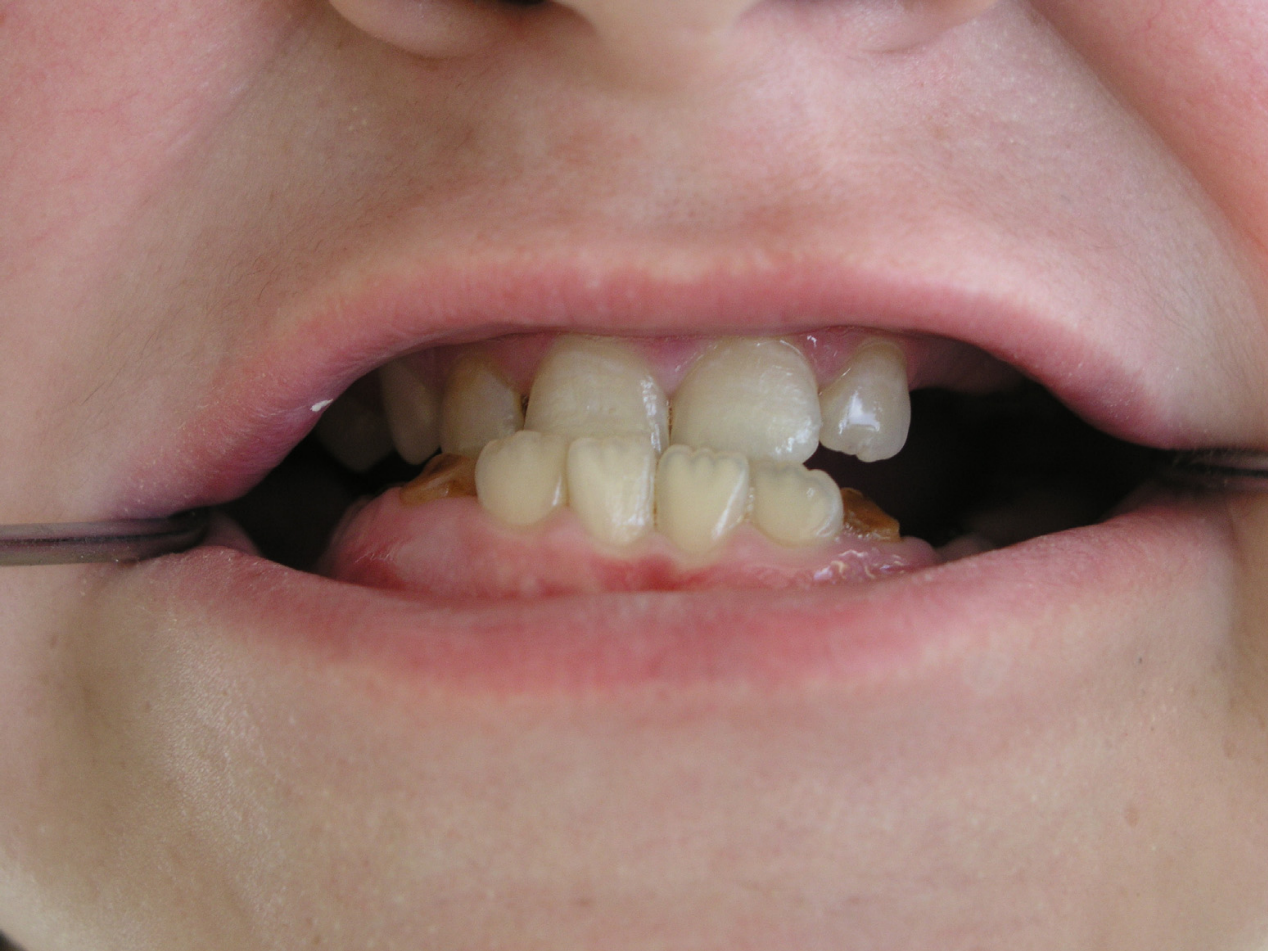
**Prognathism in a child with HIES**.

**Figure 4:**
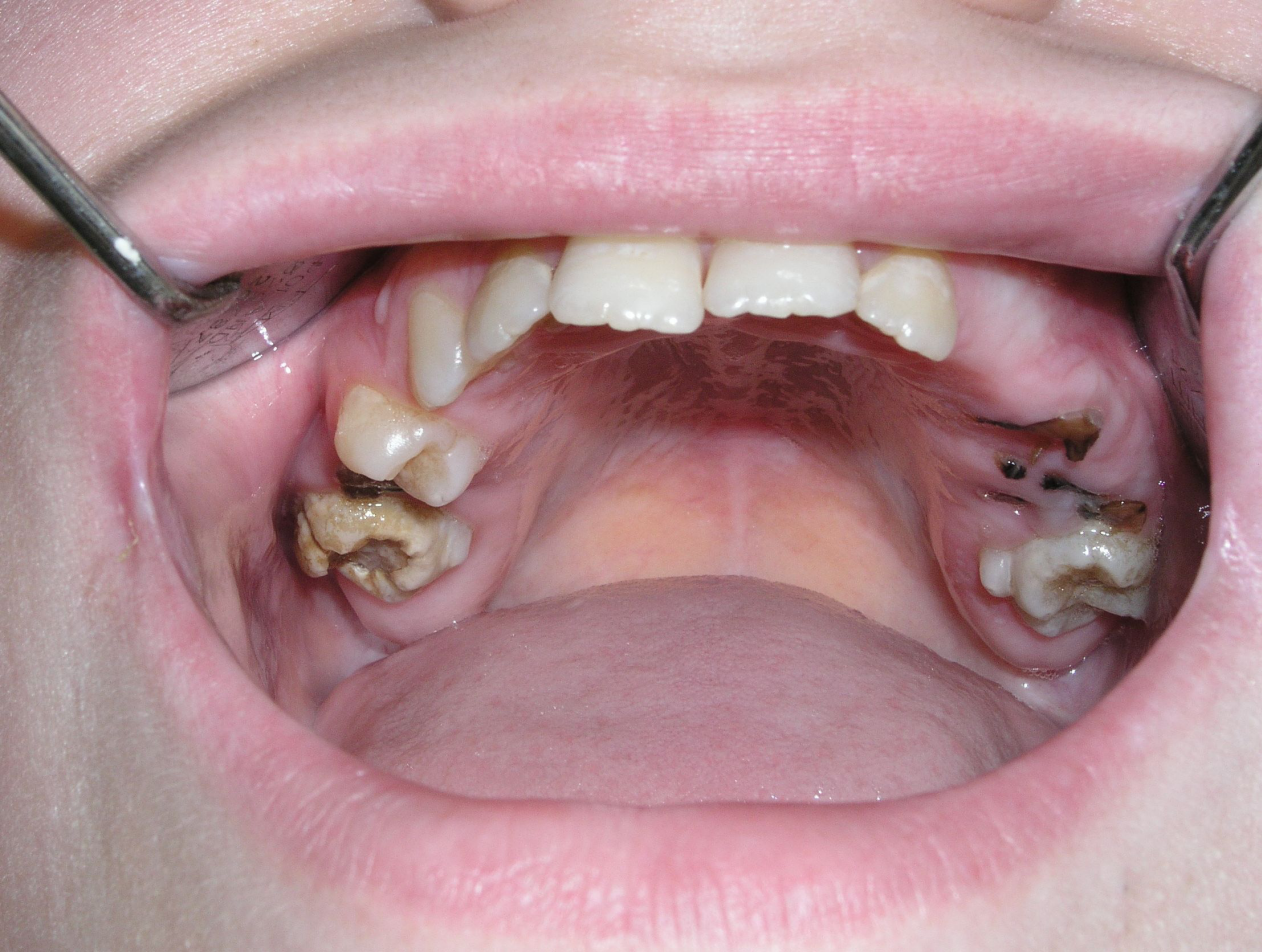
**Maxillary teeth with severely damaged primary teeth due to caries as well as carious cavities within permanent dentition in a child with HIES**.

In the majority of affected individuals, characteristic constitutional features are noticeable, such as a coarse face, rough skin, deep-set eyes, a prominent forehead, prognathism (Figure 3), thick lower lip and auricles, a wide nose and increased interalar distance [1,2,34,44,45]. There are also reports concerning mid-face anomalies, an arched palate [34] and a rare malformation - craniosynostosis [46-48]. Characteristic oral and dental manifestations occurring in HIES include the delayed loss of primary teeth, which occurred in 71% of children over the age of eight years in the study by Grimbacher et al [34], the abnormal development of permanent teeth [34,49], severe dental caries with periapical abscesses formation [50] (Figure 4) and periodontitis [51].

Recurrent pathological bone fractures reflecting the multisystemic involvement in the hyper-IgE syndrome are noted in more than 50% of patients [7]. Typically, long bones are affected [52], as well as ribs, and, less frequently, the vertebral column may also be involved [34]; these fractures occur frequently as a result of minor injuries. Brestel et al [53] described a pediatric HIES case displayng a rare association with osteogenesis imperfecta tarda. In the group of six patients in whom osteopenia was revealed initially using radiology [54] and subsequently by photon absorptiometry [55], three of them experienced pathological bone fractures. Interestingly, decreased bone mineral density is not a predisposing factor for the pathological fractures. Grimbacher et al [34] showed diminished bone density in a group of five out of nine patients, in whom densitometry was carried out, yet pathological bone fractures occurred in two patients with abnormal results of this examination and in three patients with normal bone mineral density. It has been suggested that osteopenia may result from excessive bone resorption caused by monocytes and activity of prostaglandin E2 [55]; moreover, cytokine profile in the hyper-IgE syndrome influences bone resorption, similar to women in the postmenopausal period [56]. Minegishi et al demonstrated that osteoclasts from HIES patients with *STAT3 *mutations show higher bone resorption activity compared with those from control subjects [57]. More than 60% of affected individuals present with scoliosis of varying degrees of severity and of different origin. This may be a result of different longitudinal dimension of the lower limbs, of thoracotomy for the purpose of removing pneumatocele, and of vertebral column anomalies [7], which, together with joint hyperextensibility, are another part of the hyper-IgE puzzle [46-48].

In patients with HIES, ocular complications were noted as well, including extensive xanthelasmas [58], giant chalasias [59,60], undefined eyelid nodules [61], strabismus [62], and retinal detachment with complicated cataracts [63].

The hyper-IgE syndrome brings about an increased risk of autoimmune diseases, among them systemic lupus erythemathosus [64-66], dermatomyositis [67], and membranoproliferative glomerulonephritis [68].

An increased incidence of lymphoproliferative disorders has also been noted, particularly of non-Hodgkin lymphomas [58,69-72] and of Hodgkin disease [73,74]. Oztop and colleagues diagnosed pulmonary adenocarcinoma in a patient with HIES [75].

Ling et al [76] reported coronary artery aneurysms and ectasias identified with cardiac catheterization in two patients with HIES. Subsequently, a review on the vascular features of both autosomal dominant, sporadic and recessive form of the hyper-IgE syndrome was carried out by Yavuz et al [77]. In AD-HIES patients coronary and aortic aneurysms, thrombosis of the cerebellar artery and congenital patent ductus venosus have been identified. In the report by Freeman et al [78] coronary artery tortuosity or dilation occurred in 70%, with aneurysms present in 37% of *STAT3 *mutated HIES patients, suggesting that STAT3 may play an integral role in vascular remodeling.

### Autosomal recessive HIES

Patients affected with AR-HIES associated with DOCK8 deficiency share certain clinical features with autosomal dominant HIES, such as newborn rash, eczema and recurrent upper and lower respiratory tract infections. Most of the affected patients reported by Zhang et al [79,80] suffer from recurrent otitis media, mastoiditis, sinusitis, pneumonias or bronchitis with bronchiectasis. However, parenchymal lung abnormalities, including pneumatocele formation have not been observed. Recovered pulmonary pathogens include *Streptococcus pneumoniae, Haemophilus influenzae, Pneumocystis jiroveci, adenovirus *and *respiratory syncytial virus*. Mortality is high at a young age in AR-HIES with sepsis, more frequent than in AD-HIES [9]. Furthermore, in contrast to AD-HIES, in autosomal recessive form of the syndrome, a predisposition to severe fungal and viral cutaneous infections occurs with herpes simplex and herpes zoster, molluscum contagiosum and human papillomavirus [80]. An early onset of malignancies, including cancers related to cutaneous viral infections are an important cause of death, suggesting impaired tumor surveillance. In five pediatric patients out of six recorded AR-HIES individuals, aortic and cerebral aneurysms, underperfusion of large arteries and occlusion of small cerebral vessels, variations in the caliber of the basal cerebral arteries, as well as leucocytoclastic vasculitis were diagnosed [77]. Central nervous system (CNS) sequelae, in some patients due to CNS vasculitis, such as facial paralysis, hemiplegia, ischemic infarction, and subarachnoid hemorrhages are common and contribute to high mortality. Neither skeletal abnormalities, pathological bone fractures, dental disorders, nor characteristic facial features occur in this type of HIES.

## Laboratory Findings

### Autosomal dominant HIES

A hallmark of the syndrome is an increased concentration of immunoglobulin E in the serum, exceeding 2000 U/ml, frequently higher than 5000 U/ml, and in single cases even exceeding 100 000 U/ml [5,7,34]. A value of 2000 U/ml is considered to be the cut-off point, which has proved helpful in establishing a definitive diagnosis of the syndrome [6]. Nevertheless, not in all patients, particularly in infants, are these criteria fulfilled; although characteristic concentration of IgE may be expected in the third decade of life or even later. Typically in adulthood, in a subset of patients IgE levels may decrease with age and may fall within a normal range in about 20% of cases [34]. Interestingly, the severity of infectious complications in patients with hyper-IgE syndrome do not correlate with immunoglobulin E concentration in the serum. Muhammed [81] reported on two HIES pediatric patients presenting with recurrent cutaneous lesions, severe respiratory infections and moderately elevated levels of serum IgE (420 U/ml and 564 U/ml). This further supports the view that if other features of HIES are present, a normal IgE level should not exclude the presence of HIES in older children. Concentrations of other main classes of immunoglobulins remain normal in the majority of cases. Usually blood eosinophilia coexists, wavering within values at least 2 SD above the normal range (typically higher than 700 cells/microliter) [7,22], not correlating with either the IgE concentration in the serum or the incidence of infectious complications.

In the serum, the presence of specific antistaphylococcal and anticandidal IgE antibodies may be revealed [82,83]; moreover, the increase of antigen specific antibodies may precede the first symptoms of infection [84]. However, the increased concentration of antigen specific antibodies may also be noted in patients suffering from atopic dermatitis, rendering the limitation of this test in the diagnosis of the hyper-IgE syndrome [85].

Heterogeneous disorders of the immune system have variably been described in HIES patients, including impaired production of interferon gamma by T cells, defective T helper 1 (Th1)-dependent cytokine response, a skewed Th1/Th2 cell ratio, a diminished memory T-cell populations, decreased delayed-type hypersensitivity responses, an impaired response of lymph cells to antigenic and alloantigenic stimulation [86], as well as a defective neutrophil chemotaxis [3]. However, these immunological abnormalities do not explain the unique susceptibility to particular infections seen in HIES.

In 2008, Milner and colleagues [87] demonstrated that T cells in subjects with AD-HIES failed to produce interleukin (IL)-17 (but not interferon gamma) after mitogenic stimulation with staphylococcal enterotoxin B or after antigenic stimulation with Candida albicans or streptokinase. Purified naïve T cells were unable to differentiate into IL-17 producing the T helper (Th17) cells in vitro and had a lower expression of the retinoid-related orphan receptor (ROR)-γt, which is consistent with the crucial role of STAT3 signaling in the generation of Th17 cells. These Th17 cells have emerged as an important subset of helper T cells being critical in the clearance of fungal and extracellular bacterial infections. The Th17 cytokines, IL-17 (IL-17A) and IL-17F form biologically active homo- or heterodimers. Il-17 initiates nuclear factor kappa B (NF-κB) activation, leading to the transcription of multiple target genes involved in innate immunity. These include chemokines, such as CXCL8 (IL-8) and CCL20, the cytokines IL-6, tumor necrosis factor alpha (TNF-α), granulocyte- and granulocyte-macrophage colony-stimulating factor (G-CSF and GM-CSF, respectively), acute phase proteins such as C-reactive protein, antimicrobial peptides and mucins [88]. Thus, IL-17 plays an important role in antimicrobial defenses by recruiting and expanding the neutrophil lineage and producing antimicrobial peptides. The proinflammatory cytokines produced by Th17 cells include TNFα, IL-22 and IL-26, which are involved in innate immunity, and IL-6 which directs CD4+T cells differentiation towards the Th17 lineage. IL-22 has been associated with the generation of defensins, acute phase proteins and inflammatory cytokines [89]. This multidirectional, fundamental role of Th17 cells, including cells with specificities against candidal antigens explains the pattern of infection susceptibility characteristic of *STAT3 *mutated HIES patients. In the recent report by Conti et al [90] a decrease of salivary antimicrobial peptides, such as β-defensin 2 and Histatins has been demonstrated in AD-HIES patients, providing a mechanism for the severe susceptibility to oropharyngeal candidiasis. This finding supports this hypothesis of the crucial role of the Th17-dependent responses in immunity to *Candida*.

### Autosomal recessive HIES

Immune assessments of *DOCK8 *mutated AR-HIES patients reveal T cell lymphopenia with low counts of both CD4+ and CD8+ T cells, as well as impaired T cell expansion from activated peripheral blood mononuclear cells in vitro. In autosomal recessive HIES eosinophilia and the elevated serum IgE may be more pronounced than in AD-HIES [9]. In contrast to the latter syndrome, DOCK8 deficiency is associated with low IgM concentrations and impaired generation of a durable secondary antibody response to specific antigens, which accounts for the functional antibody abnormalities [91]. In a single patient with AR-HIES due to *TYK2 *mutation, a normal number of lymphocytes was observed.

## Principles of treatment

The therapeutic strategy in hyper-IgE syndrome is directed mainly toward the prevention and management of infections. The introduction of regular long-term intake of systemic antibiotics and antifungal drugs is of great importance, as it can prevent serious, overwhelming infections and prevent lung parenchymal damage. In the empiric treatment of active respiratory infections, antibiotics introduced early on to cover such microorganisms as *Staphylococcus aureus, Haemophilus influenzae *and *Streptococcus pneumoniae *in their spectrum are recommended. Lung abscesses, which can occur frequently, particularly with staphylococcal pneumonia, may require surgical intervention. However, frequent complications after surgery also require particular attention. An important therapeutic problem as well concerns pneumatocele and bronchiectases, superinfected with *Pseudomonas aeruginosa *and other Gram negative bacteria or with fungi, such as *Aspergillus fumigatus*; in these cases conservative treatment is usually ineffective. Invasive procedures - resection of the lung parenchyma limited to pneumatocele or confined bronchiectases are high-risk therapeutic options due to the reduced ability of expanding the remaining portion of the lungs [7]. Therefore, in HIES, the decision over the resection of pneumatocele should be made with particular caution.

In contradistinction to atopic dermatitis in hyper-IgE syndrome, skin changes frequently improve only after antibiotic treatment [92]; therefore, intensive antibacterial and antifungal drugs are recommended in the therapeutic strategy of cutaneous lesions. Skin abscesses should be incised and drained. In exacerbations of eczema caused by the *Staphylococcus aureus *infection, apart from topical antibacterial treatment, emollients and corticosteroids, systemic antibiotic therapy is recommended as well. In superficial skin and mucosal candidiasis - onychomycosis, vaginomycosis and oral thrush treatment with second-generation triazole agents is effective; moreover, these drugs are also recommended in the treatment of invasive aspergillosis [93].

The use of long-term antibacterial chemotherapy, including antistaphylococcal activity in its spectrum, e.g. trimethoprime/sulfamethoxazole, semisynthetic penicillins or cephalosporins, significantly contributes to the reduction of skin abscesses and staphylococcal pneumonias [7,94]. The development of resistance in the course of long-term therapy outweighs the risk of severe infections and lung damage when it is discontinued [7]. The administration of immunoglobulins is controversial. Kimata et al [95] reported positive results of high-dose intravenous immunoglobulins, leading to the decrease in IgE concentration and in effective protection against severe infections. Even if the concentration of IgG is within the normal parameters for the age range, deficiency of IgG subclasses or lack of specific post-vaccination antibodies are observable. Consequently, the introduction of regular immunoglobulin substitution seems to be of great value; however, some authors did not note any positive effect in the use of this treatment [96]. In HIES some positive clinical effects [97], as well as an improvement of the neutrophil chemotactic function assessed in vitro [98], were achieved during prophylactic treatment with H2-receptor antagonists. Improvements in neutrophil phagocytosis and in respiratory burst were also noted with the use of sodium cromoglycate [99,100]. Poor effects of levamisole therapy reported by Donabedian [101] do not warrant its use in the case of the hyper-IgE syndrome. On the contrary, synthetic vitamin A derivative - isothretinoine proved to be effective in the treatment of cutaneous complications [102].

In vitro studies revealed an improvement in the neutrophil chemotactic function with IFN-γ [103], yet clinical observations point to the lack of a long-term effect on IgE concentration and on episodes of infection in patients with HIES [104]. Moreover, in one patient who was on interferon therapy, autoimmune thrombocytopenia was noted [105]. Reports concerning the use of cyclosporine A are encouraging; a positive clinical effect was observed by Wolach [106] and Etzioni [107]. Ishikava et al [108] and other investigators [23,109] noted an improvement after plasmapheresis. Currently, there is a lack of data associating therapeutic benefits with monoclonal anti-IgE antibody (omalizumab); the required high dose for neutralization of immunoglobulin E appears to exclude its clinical application. Attempts at hematopoietic stem cell transplantation (HSCT) also have been undertaken. Preliminary studies did not confirm the efficacy of this procedure [110,111], albeit further reports showed successful immunologic reconstitution in patients with both autosomal dominant [112] and autosomal recessive hyper-IgE syndrome [113-115]. Particularly in the latter form of HIES due to *DOCK8 *deficiency, HSCT should be considered early on before the development of life-threatening complications, including malignancies [113].

## Preliminary diagnosis

A scoring system comprising both clinical and laboratory diagnostic criteria has been proposed by Grimbacher and colleagues and accepted by the National Institute of Health (NIH) [116] (table 1). An analysis, carried out on the basis of this scale and reaching the particular total score, indicates that the affected individual is probably a carrier of the hyper-IgE genotype, or that the presence of this genotype is uncertain, or, at the very least, is less likely. However, several symptoms like scoliosis, a characteristic face, or delayed loss of primary teeth can not be taken into account in children under the age of eight, since they may not occur until adolescence. Likewise, the number of episodes with infections, bone fractures and pulmonary disorders leading to the development of pneumatocele increases with age. Therefore, in the scoring system, the age intervals of the occurrence of particular symptoms have been included. The assessment of the suspected patient according to this scoring system and the gaining ≥ 15 points makes the recognition of hyper-IgE phenotype highly probable. The diagnostic approach proposed recently by Schimke and colleagues confirmed that the NIH scoring system accurately identifies patients with HIES [117].

**Table 1:** Scoring System with Clinical and Laboratory Tests for Related Individuals with HIES

CLINICAL FINDINGS	points									
	
	0	1	2	3	4	5	6	7	8	10
**Highest serum-IgE level (IU/ml)** **[Normal < 130 IU/ml]**	< 200	200-500			501-1000				1001-2000	**> **2000
**Skin abscesses**	None		1-2		3-4				**> **4	
**Pneumonia (episodes over lifetime)**	None		1		2		3		**> **3	
**Parenchymal lung anomalies**	Absent						Bronchiectasis		Pneumatocele	
**Retained primary teeth**	None	1	2		3				**> **3	
**Scoliosis, maximum curvature**	**<**10**^0^**		10-14^0^		15-20^0^				> 20^0^	
**Fractures with minor trauma**	None				1-2				> 2	
**Highest eosinophil count (cells/μl)** **[700/μl = 1 SD, 800/μl = 2 SD]**	**<**700			700-800			> 800			
**Characteristic face**	Absent		Mildly present			Present				
**Midline anomaly (cleft palate, cleft tongue, hemivertebrae, other vertebral anomaly)**	Absent					Present				
**Newborn rash**	Absent				Present					
**Eczema (worst stage)**	Absent	Mild	Moderate		Severe					
**Upper respiratory infections per year**	1-2	3	4-6		> 6					
**Candidiasis**	None	Oral	Fingernails		Systemic					
**Other serious infections**	None				Severe					
**Fatal infection**	Absent				Present					
**Hyperextensibility**	Absent				Present					
**Lymphoma**	Absent				Present					
**Increased nasal width**	< 1 SD	1-2 SD		> 2 SD						
**High palate**	Absent		Present							
**Young-age correction**	**> **5 years			2-5 years		1-2 years		≤ 1 year		

## Genetic testing

The majority of patients with AD-HIES have heterozygous *STAT3 *mutations in the DNA-binding and Src homology 2 (SH2) domains [14,15]. All mutations are hypomorphic - missense mutations or in-frame deletions - and involve only one allele of *STAT3*, which suggests a dominant-negative effect. However, these mutations are found only in a limited number of patients with incomplete clinical features [16,57]. This observation is supported by Al Khatib et al [118], indicating molecular heterogeneity of the disease and suggesting that other potential gene defects may be functionally linked to *STAT3*. Recently, diagnostic criteria for *STAT3*-deficient individuals with clinical suspicion of HIES were proposed by Woellner and colleagues [119]. The following characteristics - total IgE concentration > 1000 IU/ml and weighted score of clinical features > 30 (based on recurrent pneumonias, newborn rash, pathologic bone fractures, characteristic face and high palate) plus a dominant-negative heterozygous mutation in STAT3 - allow for a definitive diagnosis.

Homozygous mutations in *DOCK8*, causing premature termination, frameshift, splice site disruption, single exon deletions and microdeletions were found in many, although not all, patients with autosomal recessive hyper-IgE syndrome [19]. To date, a single patient with a clinical diagnosis of AR-HIES was identified with a homozygous *TYK2 *gene mutation. This suggests that other genes may also result in the autosomal recessive form of HIES [18].

## Concluding remarks

The hyper-IgE syndrome, a multisystemic disorder with a broad constellation of clinical manifestations, is a great challenge for clinicians in establishing a diagnosis in suspected cases. HIES seems to be not a sole disease but rather a group of similar diseases with heterogeneity of underlying genetic defects. Patients with the hyper-IgE syndrome require interdisciplinary care by specialists in pediatrics/internal medicine, pneumonology, dermatology, surgery, stomatology, neurology, oncology, and psychology under the clinical immunologist's supervision.

## Competing interests

The authors declare that they have no competing interests.

## Authors' contributions

ASzP was responsible for the intellectual concept, design of the review, acquisition of data and their analysis and was the major participant in drafting the manuscript and its revision, ZK participated in acquisition of data and helped in drafting the manuscript, BP was involved in the revision of the manuscript, EHP was involved in the revision of the manuscript, AB revised critically the manuscript, KG participated in the acquisition of data and helped draft the manuscript.

All authors read and approved the final manuscript.
